# Comparison of Efficacy of Side to Side Versus End to Side Arteriovenous Fistulae Formation in Chronic Renal Failure as a Permanent Hemodialysis Access

**DOI:** 10.5812/numonthly.10248

**Published:** 2013-06-12

**Authors:** Mohammad Mozaffar, Mahtab Fallah, Saran Lotfollahzadeh, Mohammad Reza Sobhiyeh, Barmak Gholizadeh, Sayena Jabbehdari, Zeinab Mahdi

**Affiliations:** 1 General and Vascular Surgery Division, Shohadaye Tajrish Hospital, Shahid Beheshti University of Medical Sciences and Health Services, Tehran, IR Iran

**Keywords:** Kidney Failure, Chronic, Anastomosis, Surgical, Arteriovenous Fistula

## Abstract

**Background:**

In candidate patients for permanent hemodialysis or dialysis on a regular basis, an appropriate vascular access has great importance. The best permanent access is AVF (arterio venous fistula). Use of a technique to create AVF with better patency seems to be logical.

**Objectives:**

The present study aimed to compare the efficacy rate of AVFs using two different anastomosis methods; Side to Side (STS) versus End to Side (ETS) and to determine whether the different approaches render any preferences or complications.

**Patients and Methods:**

Sixty end stage renal disease (ESRD) patients were included in this clinical trial in two assigned groups of 30 patients. In one group end to side method to create AVF was used while in the other group Side to Side technique was applied for access in surgery. Both groups were followed for duration of 6 months to assess patency. For evaluating the quantitive variables, t-test was used while qualitative variables were measured using the chi-square and Fisher`s exact tests.

**Results:**

In the 6 months duration, 6 patients (20%) in the STS (side to side) group and 5 patients (16.6%) in the ETS (end to side) group experienced a non-functional AVF. In the ETS group the failure was generally a result of thrombosed access while in the STS group, 4 out of 6 patients with complications, experienced thrombosis while the other 2 patients had venous hypertension. The total failure rate was 18.3% and during the 6 months of follow up no significant difference was detected in the efficacy rate. Nevertheless, in case of longer follow ups, different outcomes could be seen.

**Conclusions:**

This study demonstrated that there was no significant statistical difference between the functional patency rates of fistulae placed by STS or ETS methods.

## 1. Background

In spite of the tremendous amounts of progress during the last decades in the field of kidney transplantation, due to reasons like high costs, unavailability of suitable donors, lack of proper care-providing centers and complications such as immunosuppression, untreatable infections or malignancies, hemodialysis still seems to be a logical and reasonable choice for treatment of candidate patients for renal replacement therapy.

A well-functioning vascular access is an important prerequisite for hemodialysis providing a repeated access to circulation with trivial complications. Vascular access complications account for 16-25% of hospital admissions in hemodialysis patients. Among the 3 principal forms of vascular access, native arterio venous fistulae (AVF) are preferred over synthetic grafts and double-lumen tunneled cuffed catheters due to their superior patency rates and lower complications. Nevertheless the high primary failure rate and maturation problems still enforce the search for better solutions (1, 2). Arteriovenous Fistula is the gold standard for vascular access for hemodialysis with an overall success rate of about 84.0%, designed to improve the effectiveness of dialysis (3).

The European guidelines recommend venous preservation and patient’s referral for access replacement 6 months prior to predicted hemodialysis. The sites being preferred for access replacement are distal arm AVF, proximal arm AVF, basillic vein transposition or graft insertion, respectively (4).

An AVF consists of a subcutaneous anastomosis of an artery to the adjacent vein. The anastomosis can be made either from the side of artery to the side of vein or from the side of artery to the end of vein. The most common AVF locations are radiocephalic (wrist), brachiocephalic (elbow), or brachiobasilic transpositions. Low resistance in the venous system leads to a substantial increase of blood flow in the artery and therefore, in the communicating vein. The artificially induced high venous blood flow leads to the dilatation of the vein and to the thickening of its wall, providing a vascular access segment that can be punctured several times a week with a large gauge needle for performing hemodialysis.

A functional AVF is described as an access that can provide a flow rate of 350-400 mL/min. The common complications of fistulae include thrombosis, venous hypertension, steal syndrome, aneurysm formation, bleeding and seroma accumulation (5).

Some mechanical factors may affect the patency of hemodialysis vascular access, such as surgical skills, puncture technique, and shear stress on the vascular endothelium. Several medical factors have also been identified to be associated with vascular access prognosis in hemodialysis patients, including stasis, hypercoagulability, endothelial cell injury, medications, red cell mass and genotype polymorphisms of transforming growth factor-B1 and methylene tetrahydrofulate reductase (2).

## 2. Objectives

The present study aimed to compare the efficacy rate of AVFs using two different anastomosis methods; side to side (STS) versus end to side (ETS) and to conclude whether the different approaches render any preferences or complications.

## 3. Patients and Methods

Sixty patients were included in this trial, who were selected randomly (simple random sample) among those referred from the Iranian dialysis center to Shohadae Tajrish hospital for hemodialysis permanent vascular access replacement during a 2 year period (2010-2012). Patients with a negative history of injection or blood sampling during the last couple of weeks and systolic blood pressure greater than 100 mmHg were selected.

Informed consents were applied. Patients were randomly divided into two groups. These groups matched, considering all demographic variables (age, gender, co-morbidities, and previously venous catheter) (Table 1, Arteriovenous Fistulae Formation2). Primarily the arteries and venous patency rates were evaluated using history and physical examination which involved arterial and venous duplex ultrasound imaging (pre and post operation), by an experienced vascular surgeon.

**Table 1.** Frequency of Patients Based on Age and Type of Treatment

Age, y	STS (%)	ETS (%)	P Value
**10-20**	2 (6.7)	1 (3.3)	0.06
**21-30**	6 (20)	1 (3.3)	0.1
**31-40**	0 (0)	2 (6.7)	0.06
**> ** **40**	22 (73.3)	26 (86.6)	0.2
**Total**	30 (100)	30 (100)	0.1

**Table 2.** Frequency of Underlying Risk Factors and Confounding Factors in the Two Groups

Groups	Diabetic Mellitus (%)	Hypertension (%)	PreviousVenous Catheter (%)
**STS**	10 (33.3)	21 (70)	16 (53.3)
**ETS**	9 (30)	21 (70)	18 (60)
**P-value**	0.06	0.08	0.06

Optimal condition of arteries and veins is crucial for access surgery. An optimal venous condition was defined as a good venous refill after its manual emptying. If superficial veins could not be visualized with a venous pressure tourniquet in place, or if any abnormality was noted on the superficial venous examination, the patient was further evaluated with a superficial venous duplex ultrasound scan. Using venous duplex imaging, superficial veins were examined for their diameter, distensibility, and continuity. The minimal acceptable diameter for use was reported to be 2 to 3 mm, whereas optimal arteries had a three plus positive pulse as an essential criterion. If any abnormality was noted on the clinical arterial examination, the patient was further evaluated with segmental pressures and a duplex ultrasound scan or pulse volume recordings. For optimal outcomes, no pressure gradient should have been noted between the bilateral upper extremities, the arterial diameter should have been greater than or equal to 2 mm throughout the extremity, and a patent palmar arch should have been present.

Dominant versus non-dominant upper extremities were chosen based on their vascultature status. Non dominant upper extremities were selected in identical conditions, while following conditions enforced AVF replacement in dominant upper extremities; unfavorable vessels, previously AVF replacement in non-dominant upper extremity, which is already out of order or same side subclavian temporary vascular access for hemodialysis. Prep and drep was done, followed by a linear incision to explore the arteries and veins of implantation site, while the peri operative systolic blood pressure was preserved at 100 mmHg.

In the STS group after obtaining control over the distal and proximal segments of the artery and vein, longitudinal arteriotomy and venotomy were performed and 10 mm side to side anastomosis was achieved. While in the ETS group, longitudinal arteriotomy was done, proximal end of the vein was anastomosed to the side of the artery using the end to side method. In cases where a good flow and thrill was not obtained, coronary dilatators for dilatation of superficial veins and evaluation of the patency of veins before anastomosis was used. For prevention of venus hypertension, distal venous ligation was performed.

Success in access surgery was defined when a good thrill was obtained whereas in the absence of thrill, hence an unsuccessful access replacement, excluded the patients from the trial. Post-surgery, a light bandage was done and all the precautionaries for early AVF thrombosis and patency preservations were explained.

All patients were required for visits on the first postoperative day, when the patency of AVF was reviewed, i.e. whether it had thrill or not and also the machinery murmur was auscultated. If any access had failed to mature, it was examined with a duplex ultrasound followed by venography if further information was necessary. The next visit was scheduled a month later and the final visit was appointed 6 months after the surgery. After initial maturation, the AV access was monitored routinely while the patient was on dialysis. The preferred method of monitoring was a monthly determination of access flow by the doppler technique. Access flow less than 600 mL/min or access flow less than 1000 mL/min that had decreased by 25% over the past 4 months were evaluated with a duplex ultrasound followed by a fistulogram if further information was necessary.

The data gathered during the 6 months follow up was registered in prepared forms and reviewed later to reach a statistical conclusion. Quantitive and qualitative data measurements were expressed by means and percentages respectively using T-test and chi-square, fisher`s exact tests.

## 4. Results

The present study included 60 patients, selected randomly from patients referred for permanent vascular access replacement surgery for hemodialysis, who were divided into two groups of 30. In one group the venous-artery anastomosing technique was end to side while in the other group the side to side anastomosing technique was used.

Among the 60 patients who entered the trial, 48 were more than 40 years old (Table 1), 19 and 42 patients had a positive past medical history of diabetes mellitus and hypertension respectively. 16 from the STS group and 18 patients from the ETS group had previous venous catheters (Table 2).

After a 6 month duration of follow-up, in 11 of the patients AVF replacement was rendered ineffective. Five of the failure cases occurred in the ETS group due to thrombosis whereas 6 cases of AVF replaced by the STS technique were unsuccessful. Failure of AVF in the STS group was explained to be a result of thrombosis and venous hypertension in 4 and 2 cases respectively.

## 5. Discussion

Our study followed 60 patients for a period of 6 months post vascular access surgery on intervals of one day, one month and finally six months after surgery. Collectively out of the 60 AVF placements using two different anastomosing techniques, 11 failed while the rest had preserved their patency.

Five out of the ineffective fistulas were in the ETS group and all were thrombosed. Six of the unsuccessful placements were in the STS group, out of which four of the accesses were thrombosed and two were non-functional due to venous hypertension. Machinary murmur without a palpable thrill was achieved immediately in one case from both groups followed by surgery. In the follow up examination a day after the surgery 2 patients in either group had failed results due to thrombosis.

In the ETS group out of the 30 cases, 4 of the fistulas were thrombosed immediately while in one case it was non-functional in the 6 months follow up. Finally on the six months follow up, in the STS group 4 cases had a thrombosed access and 2 were non-functional due to venous hypertension; whereas the total numbers of failed accesses were 5 in the ETS group due to thrombosis.

Keeping in mind diabetes as a risk factor for thrombosis, in the STS group, 10 patients were diabetic out of which two had a thrombosed access while in the ETS group 9 were diabetic, two of which experienced access failure due to thrombosis. Hypertension, also known as a thrombosis risk factor, was recorded as patient’s co-morbidity in 21 cases of the ETS group, in 3 of whom thrombosis was found as the causality of access failure.

Analysing the data for the role of sexual differences among the complicated AVF replacements have indicated the following: among 4 cases of thrombosed implants in the STS group, 3 were female opposed to the ETS group where all failed accesses occurred in male patients. 6 months follow up illustrated access failure in two cases due to venous hypertension in the STS group while those who had AVF anastomosed by the ETS technique were free of it. Generally, the failure rate in AVFs placed by side to side anastomosis technique was 20% (6 cases) versus 16.6% in the ETS group, collectively constituting the failure rate of 18.3%.

Among the unsuccessful access in the STS group, 3 out of 6 had diabetes and the others were hypertensive, while two of them were affected by both diabetes mellitus and hypertension. Similarly in the ETS group, two patients were diabetic and hypertensive and fistulae failure was seen in 3 hypertensive and 2 diabetic patients. From reviewing literature regarding the failure rate of arteriovenous fistulae, it was found that the primary failure rates were 24-35%, 9-12%, 29-36% in radiocephalic, brachiocephalic and brachiobasilic AVFs respectively (6-9).

In contrast, overall failure rate in our study was 18.3%, while the individual failure rates were 20% and 16.6% in the STS and ETS groups, respectively (10-13) which is a little higher but very much comparable to many previous studies.

Comparing the above results of the two groups with two different methods of AVF replacement during a follow up period of 6 months, we can perhaps declare that there was no significant difference in failure rates among the two groups to gain a permanent hemodialysis vascular access. Nevertheless the possibility of reaching different results with longer duration follow ups should not be ruled out and this extra follow up time will definitely help reaching a better insight regarding the AVF patency and access longevity.
